# Comparison of Cardiac Magnetic Resonance Imaging Findings and Prognostic Measures in Nondilated Cardiomyopathy and Dilated Cardiomyopathy

**DOI:** 10.1155/crp/2898685

**Published:** 2025-01-07

**Authors:** Ali Asghari, Golnaz Houshmand, Mohammad Javad Aminizadeh, Maryam Mohammadi, Sepideh Taghavi, Razieh Omidvar, Marzieh Mirtajaddini, Nasim Naderi

**Affiliations:** ^1^Cardiovascular Research Center, Rajaie Cardiovascular Institute, School of Medicine, Iran University of Medical Sciences, Tehran, Iran; ^2^Cardiovascular Imaging Research Center, Rajaie Cardiovascular Institute, Tehran, Iran; ^3^Cardiovascular Research Center, Rajaie Cardiovascular Institute, Tehran, Iran; ^4^Department of Cardiology, School of Medicine, Kerman University of Medical Sciences, Kerman, Iran

**Keywords:** dilated cardiomyopathy, nondilated cardiomyopathy, prognosis

## Abstract

**Introduction:** Nondilated left ventricular cardiomyopathy (NDLVC) is a newly defined category of cardiomyopathy. We sought to evaluate and compare the phenotype of NDLVC with DCM using cardiac magnetic resonance (CMR) imaging and to investigate the prognostic significance of these conditions.

**Methods:** One hundred and fifty patients suspected of having cardiomyopathy referred for CMR were recruited. We considered 3 groups; Group 1: NDLVC-reduced EF, (NDLVC-REF), LVEF ≤ 40%, Group 2: NDLVC-mildly reduced EF(NDLVC-MREF), 40 < LVEF < 50, Group 3: Dilated cardiomyopathy (DCM). All selected patients were followed up for a median of 24 months to determine the composite cardiac endpoint consisting of mortality and/or hospitalization for cardiovascular reasons (composite cardiac event (CCE)) as the primary endpoint.

**Results:** The mean age (SD) was 42.6 (13.7) years (range: 18–77 years). There was no association between the presence of myocardial LGE and the development of atrial and/or ventricular arrhythmias. Atrial fibrillation was most common in the NDLVC groups during the follow-up period. Myocardial late gadolinium enhancement (LGE) was also more pronounced in the DCM group. Most patients in the NDLVC groups had no LGE. LGE in the midwall was the most common LGE pattern in all three groups and the septal wall was the most commonly affected area of the LV. There was no significant difference between the CMR findings of patients with and without CCE in each subgroup. However, the presence of myocardial replacement fibrosis was higher in patients with a CCE in total study population, (*n* = 144, 68% versus 32%, *p*=0.03), but the difference was not significant in subgroup analyzes.

**Conclusion:** NDLVC has a relatively good prognosis in recent times. The consideration of NDLVC in a spectrum with DCM can be reasonable. However, the prognostic risk factors need to be investigated in more detail.

## 1. Introduction

Nondilated left ventricular cardiomyopathy (NDLVC) is a new category of cardiomyopathy recently proposed by the European Society of Cardiology [1–3]. These patients may have regional wall motion abnormality in the absence of coronary artery disease or hypokinetic left ventricle (LV) and/or LV scar without chamber dilatation. In the literature and previous studies, NDLVC is defined by an LV ejection fraction (LVEF) of less than 45% and is considered an early stage of dilated cardiomyopathy (DCM) with no significant difference in mortality between NDLVC and DCM References [1–3].However, we found that some patients with similar criteria had mildly reduced LVEF (45 < LVEF < 50) in cardiac magnetic resonance (CMR) imaging. It seems that some patients with a mild degree of left ventricular systolic dysfunction who have a nondilated heart are missing from the current definition of non-DCM. Thus, the present study was designed to further define this likely missing group of patients who appear to have NDLVC with mildly reduced LVEF. Furthermore, it would be better if NDLVC could be defined based on the current classification of heart failure syndromes, in which heart failure syndrome is divided into three categories: reduced LVEF, mildly reduced LVEF and preserved LVEF [4].

Both dilated and nondilated cardiomyopathies can lead to heart failure syndrome but differ in their structural and functional characteristics. Understanding these differences is crucial for appropriate diagnosis and management. In reviewing the clinical documents and CMR of these patients, we found that NDLVC is not simply a milder form or early presentation of DCM and that not only should the definition be better defined, but also the prognostic and outcome measures should be revised as a specific entity.

We sought to evaluate and compare the phenotype of NDLVC (both reduced and mildly reduced LVEF) with DCM using CMR imaging and to investigate the prognostic significance of these conditions in patients referred to a tertiary cardiovascular medicine center.

### 1.1. Study Population and Study Design

For the current study, 150 patients with newly diagnosed heart failure (less than 3 months) suspected of having cardiomyopathy and referred for CMR for the first time to the Imaging Department of the Rajaie Cardiovascular Institute, a tertiary center for cardiomyopathies in Tehran, Iran, were recruited between March 2019 and March 2022.

DCM defined as reduced LVEF < 50% and elevated LV end-diastolic volume indexed (LVEDVI) to body surface area (BSA), compared to published age- and sex-specific reference [5].

The NDLVC defined as (1) LVEF < 50% by CMR and (2) not elevated LVEDVI compared to same published age- and sex-specific reference values [5].

Inclusion criteria included a minimum age of 18 years, absence of coronary artery disease based on invasive selective coronary angiography or coronary CT angiography, no abnormal loading conditions (i.e., a history of severe/primary valvular heart disease, severe systemic hypertension or congenital heart disease), no active inflammation (acute myocarditis) or infiltrative heart disease, no history of chronic disease, including diabetes mellitus, chronic kidney disease, chronic lung disease. Known cases of peripartum cardiomyopathy, tachycardia-induced cardiomyopathy, arrhythmogenic right ventricular cardiomyopathy and known cases of permanent atrial fibrillation (AF rhythm) or supraventricular arrhythmias were also excluded.

The patients with DCM who had the same eligibility criteria were also selected for comparison.

The indication for CMR and its final report, demographics, clinical history and diagnosis, signs and symptoms, and medication history at the time of referral for CMR of all study populations were obtained from the patients' hospital records and by telephone calls when necessary.

### 1.2. CMR Protocol

CMR imaging was performed with a 1.5 T system (Avanto, Siemens, Erlangen, Germany) according to a standardized protocol. Late gadolinium enhancement (LGE) imaging was performed 10 min after intravenous administration of 0.15 mmol/kg gadoterate meglumine (gadolinium-DOTA, Dotarem, Guerbet S.A., Paris, France) with an inversion recovery gradient echo sequence. Images were acquired in standard long-axis planes and sequential short-axis slices (8 mm thick with a 2 mm gap) in two-phase encoding directions. Inversion times were optimized to nullify the myocardium. Ventricular volume and mass were calculated using dedicated software (CMR42, Circle Cardiovascular Imaging Inc., Calgary, Canada) and indexed to BSA.

The presence of nonischemic LGE was assessed by two independent investigators, with a third investigator making a decision if necessary. LGE was considered present if it was observed in both the long- and short-axis planes and in two coding directions and extended beyond the localized ventricular insertion areas.

### 1.3. Outcome Measures

The composite cardiac endpoint consisting of mortality and/or hospitalization for cardiovascular reasons (composite cardiac event (CCE)) was considered as the primary endpoint and the development of AF/flutter, sustained ventricular arrhythmias, and ICD implantation as the secondary endpoint.

All selected patients were followed up until the end of 2023 by reviewing their hospital records or contacting them by phone.

### 1.4. Statistical Analysis

We considered 3 groups for our study population and were able to recruit 50 patients in each group, consecutively.

  Group 1: NDLVC-reduced EF, (NDLVC-REF), LVEF ≤ 40%
  Group 2: NDLVC-mildly reduced EF(NDLVC-MREF), 40 < LVEF < 50
  Group 3: DCM

The one-sample Kolmogorov–Smirnov test was used to assess the normal distribution of the variables. Data were assessed using descriptive statistics as mean [standard deviation (SD)], median [interquartile range (IQR)], or frequency (percentage), as appropriate. For comparisons and associations, the X2 test, Student *t*-test, ANOVA, and Mann–Whitney test were used as appropriate. Adjusted associations between the CCE and other variables were analyzed using binary logistic regression multivariate analysis. The Kaplan–Meier estimator was used to represent the survival of the study population during the follow-up period. Statistical analyzes were performed using IBM SPSS Statistics for Windows, version 25.0. Armonk, NY: IBM Corp. A *p* value of 0.05 or less was considered statistically significant. The study was retrospective and conformed to the principles of the Declaration of Helsinki. It was approved by the institutional ethics committee with the ethics code IR.IUMS.FMD.REC.1402.077.

## 2. Results

In this study, 150 patients referred for CMR at the Rajaie Cardiovascular Medical and Research Institute in Tehran were examined. Six patients were excluded before final analysis because they did not meet the inclusion criteria or because their data were missing.

The mean (SD) age was 42.6 (13.7) years (range: 18–77 years). Twenty-nine (61.7%) and 52 (53.6%) patients in the DCM and NDLVC groups, respectively, were male (*p*=0.245).

Table 1 shows a comparison of baseline characteristics, clinical data and follow-up data of the NDLVC-REF, NDLVC-MREF, and DCM groups.

There was no statistically significant difference between the 3 groups in terms of age, gender, NYHA class, and family history of cardiomyopathy. Patients took beta-blockers and angiotensin-converting enzyme/angiotensin receptor blockers/angiotensin neprilysin inhibitors (ACEI/ARB/ARNI) with similar frequency, but sodium-glucose transport protein 2 inhibitors (SGLT2I) and mineralocorticoid receptor antagonists (MRA) were used less frequently, especially in NDLVC-MREF.

### 2.1. Comparison the CMR Findings

**Figure 1:**
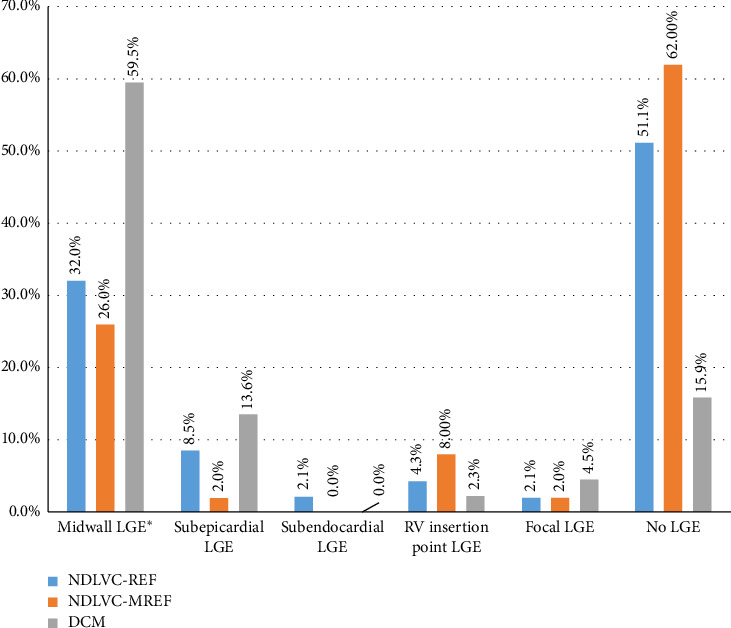
The frequency of different patterns of late gadolinium enhancement (LGE) in CMR in the subgroups of the study. ⁣^∗^Midwall LGE in septal wall, inferolateral wall, and/or other left ventricular segments. CMR, cardiac magnetic resonance imaging; DCM, dilated cardiomyopathy; NDLVC-MREF, nondilated left ventricular cardiomyopathy-mildly reduced ejection fraction; NDLVC-REF, nondilated left ventricular cardiomyopathy-reduced ejection fraction.

Table 2 and Figure 1 show the comparison of CMR findings in the subgroups of the study. As expected, right and left ventricular volumes were significantly higher in the DCM group, but there was no statistically significant difference between the three groups in terms of right ventricular ejection fraction (RVEF).

Valvular insufficiency was more severe in the DCM group. More than 85% of patients in the DCM group had mild or mild to moderate mitral and tricuspid regurgitation. Between 50% and 60% of patients in the NDLVC-REF and NDLVC-MREF groups had valve regurgitation. However, the patients with moderate and severe valve regurgitation were excluded.

Regarding the presence of LGE, myocardial LGE was also more pronounced in the DCM group. As shown in Figure 1, the pattern of LGE was different in the three groups. Most patients in the NDLVC-REF and NDLVC-MREF groups had no LGE (51% and 62%, respectively).

LGE in the midwall was the most common LGE pattern in all three groups and the septal wall was the most commonly affected area of the LV.

### 2.2. Outcome Measures

The median (IQR) follow-up period was 24 (22–36) months. During the follow-up period, the CCE occurred in 40.4%, 26% and 40.4% of patients with NDLVC-REF, NDLVC-MREF, and DCM, respectively.

The main causes of the cardiac events were symptoms related to different types of arrhythmias.

The most common symptoms were palpitations (86.2%), dyspnea (56.4%), and presyncope (18.8%). The all-cause mortality rate during the follow-up period was approximately 5% (7 out of 144). There was no mortality in patients with NDLVC-MREF. Of the 4 deaths in patients with NDLVC-REF, two patients had an automobile accident and two patients had sudden cardiac death. All 3 deaths in the DCM group were due to sudden cardiac death. The main reason for hospitalization was an arrhythmic event. AF/flutter was the most common arrhythmia in the NDLVC groups (Table 1), while the incidence of ventricular arrhythmias did not differ between the three groups.

### 2.3. Association Between Arrhythmias and Myocardial LGE

The most common arrhythmia in NDLVC population was atrial arrhythmia. In this group, myocardial LGE was found in 42% (14 of 33) of patients with AF/flutter. In addition, all 5 patients who died from cardiovascular causes had myocardial LGE.

Myocardial LGE burden was relatively high in three patients who died in the DCM group. One of them had transmural fibrosis in the midlateral, inferior and all apical segments. There was also midwall fibrosis in the basal to midseptal wall. The other two had LGE in the middle wall, both in the inferior and septal walls. The two patients in the NDLVC-REF group with SCD had LGE in the subepicardial and midinferior wall region of the LV. However, there was no statistically significant association between the presence of myocardial LGE and the development of atrial and/or ventricular arrhythmias in all study population.

**Figure 2:**
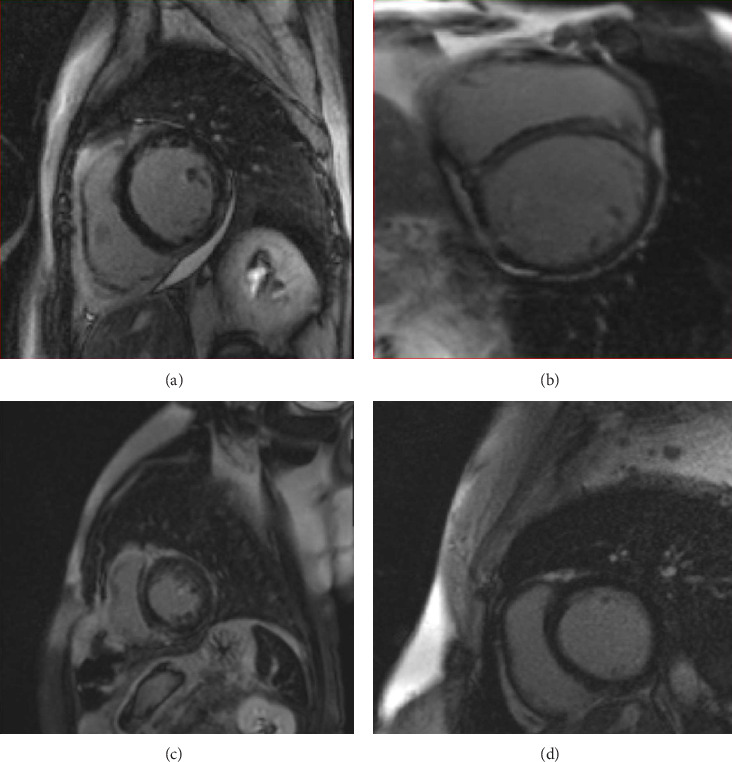
The comparison of fibrosis between two patients with DCM (a and b) and NDLVC-REF (c and d) who had SCD. These pictures show that except the size of the LV, the myocardial replacement fibrosis shown by the LGE, are seen in multiple segments of LV in both cases. The LGE burden was relatively significant in these patients which show prognostic significance of LGE and its burden in patients with different forms of cardiomyopathy. Further investigations are needed to clarify what extent of LGE and in which area of the heart could be more predictive of clinical events. (a) Short axis LGE image shows subepicardial fibrosis in the basal to midinferolateral wall in a patient with increased LV size and DCM. (b) Short-axis LGE image shows midwall fibrosis in the basal to midseptal wall in a patient with increased LV size and DCM. (c) Short-axis LGE image shows midwall fibrosis in the basal to midseptal wall in a patient with normal LV size and NDLVC. (d) Short-axis LGE image shows midwall fibrosis in the basal to midinferior wall in a patient with normal LV size and NDLVC. Abbreviations: DCM, dilated cardiomyopathy; LGE, late gadolinium enhancement; LVEF, left ventricle ejection fraction; NDLVC-MREF, nondilated left ventricular cardiomyopathy-mildly reduced ejection fraction; NDLVC-REF, nondilated left ventricular cardiomyopathy-reduced ejection fraction; RVEF, right ventricle ejection fraction.

Figure 2 shows the comparison of LGE between two patients with DCM Figures 2(a) and 2(b) and NDLVC-REF Figures 2(c) and 2(d) who had SCD.

### 2.4. Association Between CMR Findings and CCE

**Figure 3:**
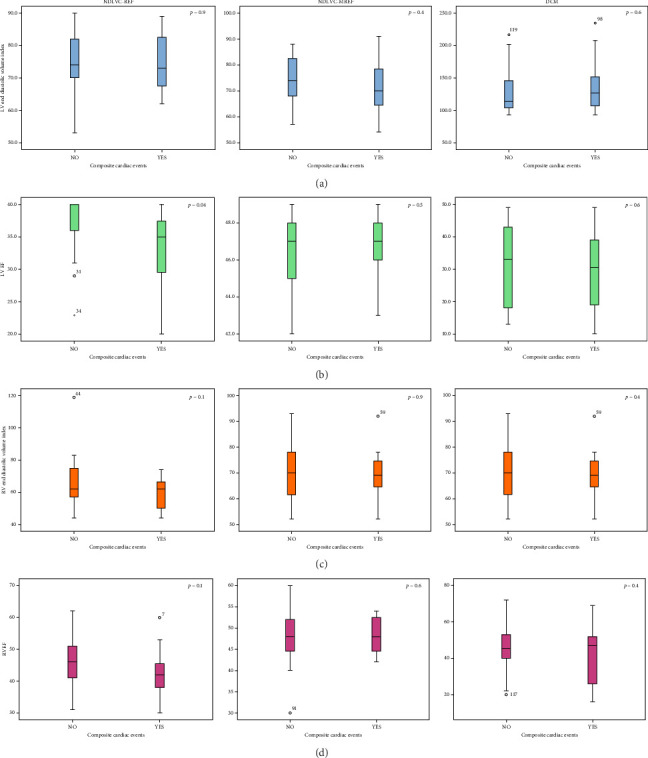
The comparison of CMR findings in patients with and without composite cardiac events (CCE) in three study subgroups. (a) left ventricle diastolic volume index, (b) left ventricle ejection fraction, (c) right ventricle diastolic volume index, and (d) right ventricle ejection fraction. Abbreviations: DCM, dilated cardiomyopathy; LGE, late gadolinium enhancement; LVEF, left ventricle ejection fraction; NDLVC-MREF, nondilated left ventricular cardiomyopathy-mildly reduced ejection fraction; NDLVC-REF, nondilated left ventricular cardiomyopathy-reduced ejection fraction; RVEF, right ventricle ejection fraction.

**Figure 4:**
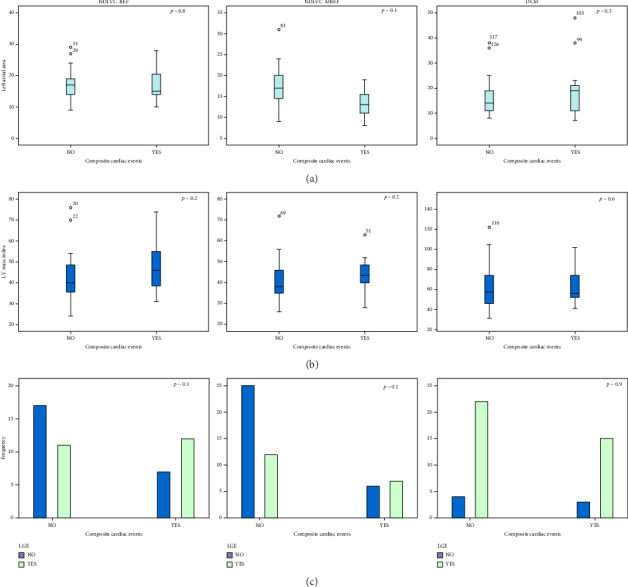
The comparison of CMR findings in patients with and without composite cardiac events (CCE) in three study subgroups. (a) Left atrial Area, (b) Left ventricle mass index, and (c) late gadolinium enhancement. Abbreviations: DCM, dilated cardiomyopathy; LGE, late gadolinium enhancement; LV, left ventricle; NDLVC-MREF, nondilated left ventricular cardiomyopathy-mildly reduced ejection fraction; NDLVC-REF, nondilated left ventricular cardiomyopathy-reduced ejection fraction.

**Figure 5:**
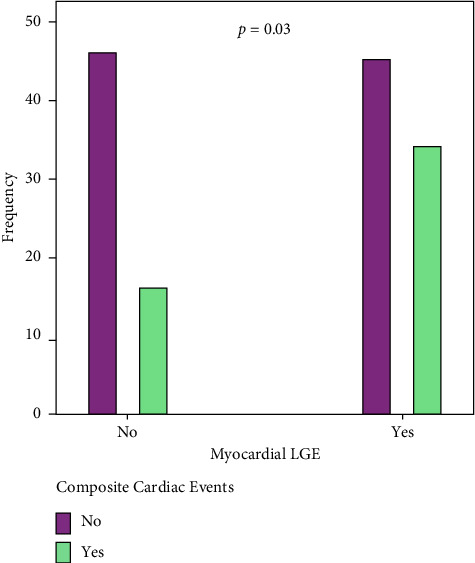
The comparison of the frequency of LGE in patients with and without composite cardiac events (CCE), *n* = 144. Abbreviations: DCM, dilated cardiomyopathy; LGE, late gadolinium enhancement; NDLVC-MREF, nondilated left ventricular cardiomyopathy-mildly reduced ejection fraction; NDLVC-REF, nondilated left ventricular cardiomyopathy-reduced ejection fraction.

Figures 3, 4, and 5 show the comparison of CMR findings in patients with and without CCE. As can be seen from the figures, there was no significant difference between the CMR findings of the patients with and without CCE.

Although the presence of myocardial replacement fibrosis was higher in patients with a CCE (68% versus 32% of the total study population, *n* = 144, *p*=0.03), as seen in Figure 5, the difference was not significant in subgroup analyzes (Figure 4(c)).

Although univariable analyzes showed no association between CCE and CMR variables, a binary logistic regression analysis (method: backward stepwise conditional) using the different CMR and clinical variables to identify independent predictors of a CCE in the entire study population (*n* = 144) showed that LVEF [beta: −0.104, *p* value: 0.007, odd ratio (95% confidence interval):0.8(0.83–0.97)] and the presence of valvular regurgitation [beta:1.64, *p* value: 0.004, odd ratio (95% confidence interval):5.1(1.67–15.97)] can be considered the strongest predictors of CCE. The results of the multivariate analyzes for each subgroup of the study are presented in Table 3. This table shows that RV end-diastolic volume index (RVEDVI) and RV end-systolic volume index (RVESVI) can be considered as the most important predictor of CCE in all three subgroups of the study.

Variables entered in step 1 of the multivariate analyzes include gender, age, myocardial LGE, LVEDVI, LVESVI, LVEF, RVEDVI, RVESVI, RVEF, LA area, LV mass index, and valvular regurgitation.

**Figure 6:**
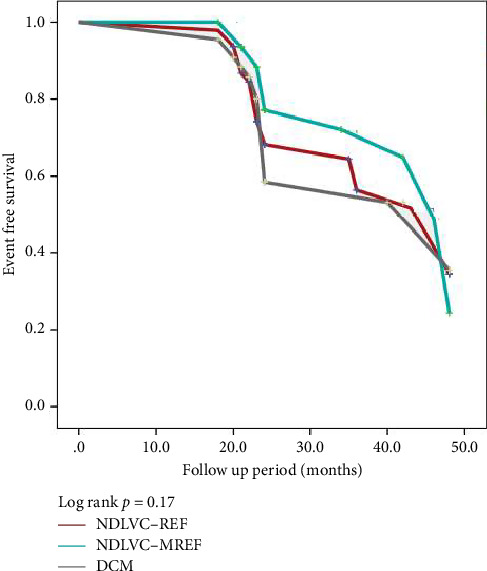
The Kaplan–Meier event-free survival curve for three study groups. Abbreviations: DCM, dilated cardiomyopathy; NDLVC-MREF, nondilated left ventricular cardiomyopathy-mildly reduced ejection fraction; NDLVC-REF; nondilated left ventricular cardiomyopathy-reduced ejection fraction.

Figure 6 shows the Kaplan–Meier event-free survival curve for our three study groups. As can be seen in this figure, the event-free survival rate of DCM patients is lower compared to the other groups, although not statistically significant.

## 3. Discussion

In the present study, we sought to describe the CMR findings and outcome of patients with NDLVC compared with DCM. Although NDLVC was defined as patients with LVEF less than 45%, we noted that some of the patients suspected of having cardiomyopathy who were referred for CMR had a nondilated hypokinetic LV with LVEF greater than 45%, so we decided to categorize our study population based on the classification of heart failure in terms of LVEF.

We endeavored to carefully select our study population and select idiopathic cases. Thus, all patients with underlying diseases or abnormal loading condition which might affect the cardiac function were excluded. In addition, our study population was selected from newly diagnosed cardiomyopathies referred for further CMR investigation. All of these patients were clinically managed by a dedicated multidisciplinary cardiomyopathy treatment team and received heart failure guidelines directed therapies.

Patients with abnormalities of LV ejection fraction, wall motion or LV scars without LV dilatation are diagnosed with NDLVC. Although this recently defined group of cardiomyopathies is categorized in the DCM spectrum due to similarities with DCM, there are many differences and controversies in this regard [1, 6–12].

We think that the main reason for the contradictory results of the different studies lies in the definition and categorization of this type of cardiomyopathy based solely on the morphological features of the LV. Moreover, the differences in the study population and clinical background of the various studies could be the reason for these discrepancies. Our study showed similarities and differences with previous studies comparing DCM and NDLVC. Although some limitations of retrospective studies need to be considered in the current study, the results highlight some important points.

### 3.1. Comparison of CCE in DCM and NDLVC

Some studies have shown that long-term outcomes may be worse in patients with DCM compared with NDLVC [8], other studies [6], suggest that the midterm outcomes of patients with DCM are similar to those of patients with normal or mildly dilated LV, even after adjustment for LVEF and comorbidities. We had a midterm follow up and found a trend toward lower event-free survival in patients with DCM (Figure 6); however, this comparison was not statistically significant in terms of CCE. It seems that a longer follow-up period is needed for more precise results.

Another important issue in studies of CMR findings in cardiomyopathies is the presence and burden of LGE, which indicate myocardial replacement fibrosis and have prognostic significance. LGE burden was higher in the DCM group, which was expected given the prognostic significance of the presence of LGE in CMR. Eda et al. found that DCM patients had a higher prevalence of LGE, a sign of more severe myocardial fibrosis, than patients with NDLVC-REF [6].

There are several studies showing that multiple LGE lesions are independent predictors of cardiovascular events in patients with nonischemic cardiomyopathy, including mortality, ventricular arrhythmias and sudden cardiac death, hospitalization for heart failure, and transplantation during long-term follow-up. In this study, the presence of myocardial replacement fibrosis was higher in patients with a CCE (Figure 5), fibrosis burden was more severe in patients who died, and 42% of patients with atrial arrhythmias in the NDLVC group had LGE. Furthermore, patients with NDLVC-MREF who had a higher LVEF were more likely to have a lower myocardial LGE burden and a better clinical outcome. However, there were no significant differences between the three groups in terms of the CCE.

Apart from the shorter follow-up time of our study population, a possible explanation for the current outcome could be that our patients did not have many risk factors for a worse outcome, including diabetes mellitus, systemic hypertension, significant valvular heart disease or systemic diseases. In addition, compared with previous studies, our patients were newly diagnosed, were younger, and were all treated at a tertiary center for cardiomyopathy in the fantastic four era, [2], which may also result in less disease progression and mortality. This finding emphasizes the importance of earlier initiation of heart failure-specific medical therapy to reduce the progression of cardiomyopathy and improve outcomes. Furthermore, there is currently no consensus on what extent of LGE is predictive of clinical events, as there is no standardization for defining the extent of LGE and the definition of ventricular arrhythmias varies between studies seeking to correlate LGE with ventricular arrhythmias. For example, some studies have included ventricular arrhythmias that are not life-threatening, such as PVCs and nonsustained ventricular tachycardia, in their composite endpoints [13].

### 3.2. Importance of Atrial Arrhythmias and Sudden Cardiac Death in NDLVC

The presence of a high incidence of atrial arrhythmias in NDLVC group may be a sign of tachycardia-induced cardiomyopathy, especially in patients without LGE [14]. Therefore, simply defining a patient as having a nondilated form of DCM may not take into account the likelihood of developing AF and other complications associated with AF. Therefore, it is advisable to pay special attention to these patients, perform a thorough CMR examination to detect the presence of LGEs and further monitor them for AF [15].

In addition, although the patients with arrhythmogenic RV cardiomyopathy were excluded in this study, incident SCD and ventricular arrhythmias could be signs of arrhythmogenic LV cardiomyopathy in some patients.

### 3.3. Familial Predisposition

The positive family history of cardiomyopathy in 18.5% of patients with NDLVC suggests that genetic studies are needed in this patient group to find better predictors of mortality and morbidity and to search for further personalized treatment options.

Eda et al. suggested that the frequency of AF (AF rhythm) and increased LV filling pressure might be risk factors for adverse events and make the risk of death in NDLVC-REF and DCM comparable [6].

Another point of our study that should be considered is the absence of congestion-related cardiac events. Although dyspnea was the main cause of hospitalization in some patients, arrhythmias were the main cause of CCE in this study, and the majority of patients were hospitalized for an arrhythmic event. The higher rate of arrhythmias in these patients may indicate a type of atrial cardiomyopathy that needs to be investigated in further studies.

Furthermore, it seems that we need to take a second look at the natural history of many types of nonischemic cardiomyopathies when applying current medical therapies for heart failure syndromes.

### 3.4. Study Limitations

The careful selection of the study population and the existence of a follow-up period can be considered strengths of the current study. Since we included newly diagnosed patients, it would be better if we could follow them up over a longer period of time. On the other hand, since we excluded patients with comorbidities, the generalizability of the study results might be limited, especially in the group of patients who have NDLVC and systemic diseases such as diabetes mellitus and connective tissue diseases.

Although we did not collect echocardiographic findings in this study, the selection of patients based on CMR criteria could be a strength of the study, as CMR findings such as size and EF are considered the gold standard compared to echocardiography.

It would be better if we could compare the echocardiographic data at the beginning and after treatment to show the remodeling of the myocardium.

Another limitation could be the lack of laboratory findings, especially the absence of natriuretic peptides. We tried to select patients who were otherwise normal and had only cardiomyopathy, so their renal and liver function and cell blood count were within normal limits. Regarding natriuretic peptides, unfortunately in our country we have limited ability to check the levels of probrain natriuretic peptide or brain natriuretic peptide, and many patients did not have natriuretic peptide checked at the time of performing CMR.

Also, we wanted to recruit more patients, but we had COVID-19 during the study period, so despite the high volume of our center for performing CMR, we could only recruit 150 patients during the study period. Further studies or inclusion of the results of the current study in secondary studies should be conducted to elucidate subtle differences in different subgroups of cardiomyopathies with reduced ejection fraction.

In conclusion, it can be said that NDLVC currently has a relatively good prognosis. Consideration of NDLVC in a spectrum with DCM may be reasonable. In this spectrum, there are some patients with NDLVC who have mildly reduced EF who may have a better prognosis and should not be ignored. Thus, the conflicting results of different studies on NDLVC show that this type of cardiomyopathy and its prognostic risk factors need to be further investigated.

## Figures and Tables

**Table 1:** Comparison of the baseline characteristics, clinical data, and follow-up data of the NDLVC-REF, NDLVC-MREF, and DCM groups.

Variables	NDLVC-REF *N* = 47	NDLVC-MREF *N* = 50	DCM *N* = 47	*p* value
Age, years, mean (SD)	43.0 (13.1)	39.4 (11.8)	44.0 (15.8)	0.428

Sex, male, *n* (%)	24 (51.06)	28 (56%)	29 (61.7)	0.24

NYHA class, *n* (%)				0.14
I	15 (31.9)	16 (32)	14 (29.7)	
II	26 (55.3)	27 (54)	25 (53.2)	
III	6 (12.8)	7 (14)	8 (17.02)	

Family history of cardiomyopathy, *n* (%)	10 (21)	8 (16)	8 (17)	0.7

Atrial flutter/fibrillation, *n* (%)	20 (42.5)	13 (26)	7 (14.9)	0.04

Sustained ventricular arrhythmias, ICD implant, *n* (%)	8 (17)	5 (10)	8 (17%)	0.2

*Baseline medical therapies*				
Beta blocker, *n* (%)	46 (97.9)	44 (88)	45 (95.7%)	0.2

ACE.I/ARB/ARNI, *n* (%)	46 (97.9)	40 (80)	45 (95.7%)	0.29

SGLT2I, *n* (%)	21 (44.7)	15 (30)	41 (32.6%)	0.05

MRA, *n* (%)	40 (85.2)	28 (54)	35 (81.4%)	0.01

Diuretics, *n* (%)	2 (4)	1 (2)	30 (63.8)	< 0.001

GDMT at follow up^$^	46 (97.8)	40 (80)	42 (89.3)	0.0001

Composite cardiac event	19 (40.4)	13 (26)	19 (40.4)	0.22⁣^∗^ 0.05⁣^∗∗^

All-cause mortality	4 (8)^#^	0	3 (6)	0.041

Abbreviations: ACEI, angiotensin-converting enzyme; ARB, angiotensin receptor blockers; ARNI, angiotensin neprilysin inhibitors; DCM, dilated cardiomyopathy; GDMT, guideline directed medical therapy; MRA, mineralocorticoid receptor antagonists; NDLVC-MREF, nondilated left ventricular cardiomyopathy-mildly reduced ejection fraction; NDLVC-REF, nondilated left ventricular cardiomyopathy-reduced ejection fraction; NYHA, New York Heart Association; SGLT2I, sodium-glucose transport protein 2 inhibitors.

^#^The cause of death was noncardiac (car accident) in two patients.

^$^Heart failure guideline directed medical therapy using fantastic four (ACEI/ARB/ARNI + beta blockers + MRA + SGLT2I).

⁣^∗^*p* value of comparison between the 3 groups.

⁣^∗∗^*p* value for comparison between NDLVC-MREF and DCM or NDLVC-REF.

**Table 2:** Comparison of the CMR findings in study subgroups, *n* = 144.

Variables	NDLVC-REF *N* = 47	NDLVC-MREF *N* = 50	DCM *N* = 47	*p* value⁣^∗^
Left ventricular mass index, mean (SD)	44.5 (11.4)	41.9 (9.3)	62.7 (22.4)	< 0.001
Left atrial area total, mean (SD)	17.2 (5.9)	16.2 (4.5)	17.0 (8.6)	0.87
Left ventricular end-diastolic volume index mean (SD)	74.9 (9.2)	73.7 (9.5)	130.7 (36.5)	< 0.001
Left ventricular end-systolic volume index mean (SD)	48.1 (7.6)	39.5 (5.02)	91.3 (41.0)	< 0.001
Left ventricular ejection fraction mean (SD)	35.6 (5.6)	46.5 (1.9)	32.4 (13.0)	< 0.001
Right ventricular end-diastolic volume index mean (SD)	63.5 (1.4)	70.4 (10.1)	91.0 (29.7)	< 0.001
Right ventricular end-systolic volume index mean (SD)	34.7 (8.7)	36.3 (6.1)	52.9 (23.3)	< 0.001
Right ventricular ejection fraction mean (SD)	44.6 (7.7)	48.08 (5.3)	44.2 (13.9)	0.301
Mitral and tricuspid regurgitation, *n* (%)	26 (55.3)	28 (56)	41 (87)	0.006
Myocardial LGE (Fibrosis), *n* (%)	23 (49)	19 (38)	38 (80.8)	0.0005

Abbreviations: CMR, cardiac magnetic resonance imaging; DCM, dilated cardiomyopathy; NDLVC-MREF, nondilated left ventricular cardiomyopathy-mildly reduced ejection fraction; NDLVC-REF, nondilated left ventricular cardiomyopathy-reduced ejection fraction; SD, standard deviation.

^∗^The significance level of *p* value is < 0.05.

**Table 3:** Independent predictors of a composite cardiac event in each study subgroup, *n* = 144.

Variables	Beta	*p* value	Odd ratio (95% CI)
*NDLVC-REF, n = 47*			
RV end-systolic volume index	0.19	0.02	1.21 (1.02–1.43)
LV Mass index	0.08	0.03	1.08 (1–1.2)

*NDLVC-MREF, n = 50*			
RV end-diastolic volume index	0.28	0.04	1.33 (1.01–1.76)

*DCM, n-47*			
RV end-systolic volume index	0.05	0.007	1.05 (1.01–1.09)

Abbreviations: CI, confidence interval; LV, left ventricle; NDLVC-MREF, nondilated left ventricular cardiomyopathy-mildly reduced ejection fraction; NDLVC-REF, nondilated left ventricular cardiomyopathy-reduced ejection fraction; RV, right ventricle.

## Data Availability

The data that support the findings of this study are available on request from the corresponding authors. The data are not publicly available due to privacy or ethical restrictions.
